# St. James's Infirmary

**Published:** 1832-08

**Authors:** 


### HOSPITAL REPORTS.

### ST. JAMES'S INFIRMARY.

*Case, illustrative of the Contagious Propagation of Cholera.* Communicated by ALFRED S. TAYLOR, Lecturer on Medical Jurisprudence in Guy's Hospital.

THE controversy which has been for so long a period kept up in the profession, regarding the contagious propagation of a disease with which we have had, unfortunately, but too frequent opportunities of making ourselves acquainted, has now for the most part subsided, and it might appear both futile and unnecessary to revive it. Still where a fact presents itself, which may seem to throw some light upon the obscurity which has often marked the progress of the disorder, it is well that it should be recorded; for I will not presume that any member of our profession, whether contagionist, anti-contagionist, or neutral, has any other desire than that of arriving at the truth. The appeal to facts, which has been uniformly made by all parties, has failed in producing conviction on the minds of those who were, from the first, opposed in their views; a result which others, who are not in the profession, have not failed to take the advantage of; and hence have arisen the abuse and calumny which have been heaped upon certain members of our profession, for the maintenance of opinions that probably the lapse of time will prove to have been well founded. Notwithstanding this, it can only be by *an appeal to facts* that the question will ever arrive at a settlement; but I am willing to admit that these have often been so ambiguous as to leave the question in the same undetermined state in which it had previously existed. I do not pretend that the fact which I have to report is so peculiar as to be free from all objection; nor do I believe that any such will readily be found, since, as long as there exist two opinions upon the same subject, it is natural to expect that two or more explanations will be adduced; the value of which, however, it is for the profession, and not for any single individual, to determine.

Mrs. Birke, a middle-aged woman, the matron of the St. James's Infirmary, was employed in sitting up with a recently admitted cholera patient, on Saturday, July 7th; and again, upon the morning of Sunday, July 8th, her services were required for a child that had just been brought in, labouring under the same disease. She assiduously attended to these two cases in the absence of the nurse, and remained by the bedside of her last patient until she herself complained of feeling unwell. This was about three hours after her attendance upon the child, and about fifteen hours from her first attendance upon the female. The disease rapidly manifested itself in her: she soon passed into the stage of collapse, and, in spite of all the means employed to subdue the violent progress of the symptoms, she died in eleven hours from the time of her seizure. I may add, that the female whom she first attended died, but the child eventually recovered.

A patient investigation of this case has led me to consider it to be a decided instance in which the cholera was received by a communication with the infected; and my reasons for believing it to have been such I will now proceed to state. Mrs. Birke, up to the moment of her coming in contact with the female admitted on the Saturday evening, had been free from every appearance of disease, and in the early part of the day had employed herself in her usual occupations, her duties not allowing her to leave the infirmary. She was regular in her habits, and, indeed, her situation, as matron to the establishment, would place her above the suspicion of being given to those practices which many of the lower order of nurses indulge in. I make this statement merely to shew that the disorder had not manifested itself in her by any premonitory symptoms; for any error in her general habits would merely have reference to the *predisposition* of her system, and not to the *actual production* of the disease. It is important to remark, that the street in which the infirmary is situated has, as far as can be ascertained, remained entirely free from cholera, the cases which have presented themselves in it having been brought from other parts of the parish. Now, I think, from these premises, we have a fair right to assume that, if the two individuals labouring under cholera had not been introduced into the infirmary, Mrs. B. would not have had the disease. I am at a loss to conceive how this case can be explained in any other way, unless by violating all those common rules of reasoning which apply to other diseases that are acknowledged by all to be communicable. To say that it is to be explained by supposing the malady to be purely epidemic, appears to me to be altogether unwarrantable. The smallpox may rage epidemically, and individuals may be seized without any PROOF of their having come in contact with the infected; but if these persons had had the smallpox instead of the cholera, supposing the former to be equally as prevalent as the latter is now, and the nurse or matron, after unremitting attendance on the patient, had been seized, and had perished in consequence, would it not be considered absurd to contend that the latter had taken it through an epidemic state of the atmosphere, and not through personal exposure to the infected? But it is said smallpox is known to be personally communicable. This is certainly true; but how has this been ascertained? I reply, only by a *multiplication of facts* of the description of that which I have adduced.

It is not a little remarkable that the majority of those who have treated of this subject should have contented themselves with *simply denying* that the cholera is to any degree contagious or communicable by contact or proximity: they have never ventured to assign a reason why this should not be one of the means of its propagation, although one would suppose that some such reason must have been apparent to themselves. On the contrary, they have not only not done this, but they have ventured to stretch the doctrine of probability to an extreme to which few, perhaps, of the unprejudiced would be disposed to follow them. They have brought forward hypotheses far more vague than the doctrine of contagion; and because the latter is incapable of being reconciled with every fact observed in the progress of the cholera, it has been hastily asserted that the disease is not in any degree contagious. There will, no doubt, be many to whom the reasoning in this single instance will not bring home conviction; although it appears to me to be so clear a catenation of cause and effect, that, if it referred to any other phenomenon in nature, it would at once be considered conclusive; and if, instead of once only, it occurred a thousand times, what would be the verdict of an impartial jury in such a case? Yet it should be remembered the *repeated* occurrence of such cases can merely have reference to the *degree* of communicability of the disease, and not to its *actually contagious nature*. Now, according to the views of non-contagionists, it would matter not how often such cases were observed; for it is manifest if *one case* were to be explained away by supposing the individual to have received the germ of disease from the air, (or any other undemonstrable source,) the *whole thousand* must be explained away in the same manner; or, in other words, if we do not imagine that the disease has been received by proximity to the sick in one instance, we cannot admit it, even although it were to occur a thousand times in succession. Does this not shew that that which is required to be proved is absolutely assumed? namely, that the promiscuous intercourse between individuals labouring under cholera has nothing whatever to do with the diffusion of the malady. It is a well-known fact, that individuals in the same house, in the same street, or in the same quarter, are now, before our eyes, successively attacked: we are told that such cases are to be considered as not having any relation to each other with regard to contagious communicability; but that they proceed from a vitiated state of the air, and that each would occur independent of all intercourse. But what are the *proofs* upon which such an assertion rests? How has it been ascertained that the number of such cases is *not augmented* through personal intercourse? The only answer to these queries that I have met with is this, "that the cholera has appeared in places where no intercourse has been proved to have taken place, and has not subsequently spread." I would not reply to such an argument by saying, that the same has been remarked not only with regard to cholera, but with regard to every other contagious disorder; even the smallpox, although this would be sufficient to display its futility: but I will take it upon another ground; namely, that it involves a gross fallacy in reasoning. It absolutely assumes that facts are of the nature of acids and alkalies; i. e. that they *neutralize* each other, and form a *tertium quid*, which does at once away with the whole theory of contagion? For instance, if a case like that of Mrs. B. be adduced, in which contagion is rendered more than probable, it is opposed by saying that another instance has occurred in which there was no disease propagated, or, as might be said in the case of Mrs. B., *both the nurse and the surgeon escaped:* but would it not be an outrage upon common sense to affirm that, because A and C did not receive the disease through personal contact, B could not have *thus* received it. Yet this is the very substance of all the arguments brought forward against the contagion of cholera; and upon such an assumption, upon such a violation of all inductive reasoning, we are called upon to affirm, that, in cases where intercourse has been known and proved to have existed, *that intercourse* had nothing whatever to do with the propagation of the disorder. One would suppose that we had returned to the olden time, when the earth was considered to be a level plain, and the moon held to be no larger than Peloponessus; when hypothesis took the place of theory, and the dogmas of Aristotle passed for laws of nature; for assuredly the reasoning adopted by some with regard to the non-contagious propagation of cholera, is only to be reconciled with the supposition that we are living in the infancy of science, and that the great Bacon, the "*minister et interpres naturæ*," has passed through the world to no purpose. That the cholera has appeared in places where no intercourse has been *proved*, is most true: that in *hundreds* of *instances* it has not spread from the focus of its appearance, *may also be true:* but such *negative* facts can never do away with others of an *affirmative* nature: they may teach us that it is not in every case contagious or liable to spread, conditions which have been long since known to and admitted by the observers of its progress; but how can they justify the inference, that the disease is *never* communicated by contagion?

I do not know how the recent appearance of the disease in our settlements in Canada is to be explained away, or to what hypothesis those who deny the possibility of contagion being imported will resort: but I think it will be difficult to convince either the profession or the public that the breaking out of the cholera in Quebec had no relation whatever with the arrival of emigrants from an infected country, when it is known that many of our ships have been compelled to put back with the cholera on board. Possibly it may be said that ships have left this country for other parts, and yet the disease has not appeared in these cases; and we may be called upon to admit that, because the disease has not *always* been exported, it can in no case have been exported; but that it must have taken its origin from some other cause? If such a principle be once allowed to influence the opinion which we may form, we may safely say that the question resolves itself into nothing; all hopes of imparting conviction with regard to what is called contagion, must be abandoned, and all necessity for it is at once set aside!

I have been led into a somewhat longer discussion upon this subject than I had intended; but I will conclude this paper by stating that, to my mind, the case of Mrs. Birke proves most clearly that the cholera is occasionally (perhaps more universally than is imagined) communicable from one individual to another; and to resort to any other explanation in her case, appears to me to be as superfluous as to suppose that an individual, who has voluntarily exposed himself to the fumes of carbonic acid, should have died from any other cause than from the noxious influence of that gas.

